# A non-invasive nomogram for high-risk esophageal varices in cirrhosis without transient elastography: development and internal validation

**DOI:** 10.3389/fmed.2026.1946799

**Published:** 2026-09-18

**Authors:** Yi Wu, Junyang Luo, Shuixian Yang, Shuaishuai Kong, Dandan Chen, Wenhui Guo, Nianfeng Fan, Zhebin Wu, Yurong Gu, Zhaoxia Hu, Zhiliang Gao, Yubao Zheng

**Affiliations:** 1 Department of Infectious Diseases, The Third Affiliated Hospital of Sun Yat-sen University, Guangzhou, Guangdong, China; 2 Department of Infectious Diseases, Zhaoqing Hospital, The Third Affiliated Hospital of Sun Yat-sen University, Zhaoqing, Guangdong, China; 3 Department of Interventional Radiology, The Third Affiliated Hospital of Sun Yat-sen University, Guangzhou, Guangdong, China; 4 Department of Information Technology, The Third Affiliated Hospital of Sun Yat-sen University, Guangzhou, Guangdong, China

**Keywords:** high-risk esophageal varices, liver cirrhosis, nomogram, non-invasive model, portal hypertension

## Abstract

**Background:**

Gastroesophageal varices (GOV) and variceal hemorrhage are major life-threatening complications of cirrhosis. Although esophagogastroduodenoscopy (EGD) remains the diagnostic gold standard, its invasiveness, cost, and limited patient adherence underscore the need for reliable non-invasive risk-stratification tools. We aimed to identify independent predictors of high-risk esophageal varices (HREV) and develop and validate a simple, cost-effective nomogram integrating routinely available clinical and ultrasonographic parameters.

**Methods:**

In this retrospective cohort study, we enrolled 575 cirrhotic patients hospitalized between January 2010 and December 2019. All participants underwent contemporaneous EGD and abdominal color Doppler ultrasonography during the same hospitalization. A nomogram was constructed based on independent predictors identified by multivariate analysis. Model performance was evaluated using the area under the receiver operating characteristic curve (AUROC), calibration curves, decision-curve analysis (DCA), and compared with established non-invasive scores (APRI, AAR, FIB-4, PC/SD) and with each individual predictor; the training-derived threshold was fixed and applied unchanged to the validation cohort.

**Results:**

Endoscopy identified HREV in 71.8% (413/575) of patients. Multivariate analysis revealed six independent predictors: male sex, hemoglobin (HGB), platelet count (PLT), right liver thickness (RLT), portal vein diameter (PVD), and spleen diameter (SD). The developed nomogram demonstrated satisfactory discrimination, with AUROCs of 0.803 (95% CI: 0.755–0.850) in the training cohort and 0.807 (95% CI: 0.734–0.880) in the validation cohort, outperforming the biochemical scores APRI and AAR, and significantly improving risk reclassification over both FIB-4 and the platelet count-to-spleen diameter (PC/SD) ratio (NRI and IDI, all *P* < 0.05). It also outperformed each single component in the training cohort (spleen diameter alone, 0.742; *P* = 0.003) but not in validation (*P* = 0.061); at the fixed threshold of 0.770 the negative predictive value was 48.2%. Calibration and DCA confirmed satisfactory predictive accuracy and clinical utility.

**Conclusions:**

A simple nomogram based on routine clinical and ultrasonographic variables stratified HREV risk and outperformed established biochemical scores. Its negative predictive value in this high-prevalence cohort was insufficient to defer endoscopy safely; the nomogram is therefore proposed for prioritising endoscopy where transient elastography is unavailable, rather than as a substitute for Baveno-based rule-out strategies. External validation in lower-prevalence populations is required.

## 1 Introduction

Cirrhosis represents a substantial global health burden, representing the end-stage of chronic liver disease (1, 2). In China, hepatitis B virus (HBV) infection remains the predominant etiology, driving the natural history from an asymptomatic compensated stage to a decompensated stage marked by complications (3, 4). Portal hypertension (PH), a pathological hallmark of this progression, leads to the formation of gastroesophageal varices (GOV) in approximately 50% of patients (2, 5, 6). Among these, high-risk esophageal varices (HREV), defined as medium-to-large varices (≥5 mm), varices of any size with red color signs, or small varices in patients with Child-Pugh class C, are particularly critical (5, 7). Because HREV is associated with an annual bleeding risk of 15% and a mortality rate of up to 40%, current guidelines recommend immediate primary prophylaxis with non-selective beta-blockers (NSBBs) or endoscopic ligation once HREV is identified (5, 7–9).

Currently, hepatic venous pressure gradient (HVPG) measurement and esophagogastroduodenoscopy (EGD) are the diagnostic gold standards (5, 10). However, HVPG is invasive and technically demanding, restricting its use to specialized centers. While EGD is definitive, its invasiveness, high cost, and potential procedural risks (e.g., aspiration or arrhythmia) often lead to poor patient compliance (11–13). To mitigate this, the Baveno VI criteria proposed using liver stiffness measurement (LSM) via transient elastography (TE) combined with platelet counts to rule out HREV (7), concepts that were reaffirmed and refined in the recent Baveno VII consensus (14). However, TE equipment is not universally available in resource-constrained settings or grassroots hospitals, and its accuracy is often compromised by obesity, narrow intercostal spaces, or ascites (15–18). More importantly, TE devices are costly and require regular calibration, making them inaccessible in many rural or primary healthcare facilities in developing regions. Furthermore, traditional serum-based scores (e.g., APRI, FIB-4) have shown limited sensitivity for HREV prediction (19).

Consequently, there is an urgent need for a risk stratification tool that relies solely on ubiquitously available parameters. Although Doppler ultrasonography is inexpensive and widely accessible, a comprehensive model integrating it with routine blood markers is lacking. Therefore, this study aimed to identify independent risk factors for HREV and develop a practical, simple nomogram based on standard clinical and ultrasonographic variables. The intended purpose was defined before the analysis: to rank patients by their probability of harbouring HREV so that limited endoscopic capacity can be directed to those most likely to benefit. The model was not designed, and is not proposed, as a means of excluding varices without endoscopy.

## 2 Methods

### 2.1 Study design and participants

This retrospective cohort study was conducted at the Third Affiliated Hospital of Sun Yat-sen University. We screened 1,250 cirrhotic patients hospitalized between January 1, 2010 and December 31, 2019. Inclusion criteria were: (1) age >18 years; (2) diagnosis of cirrhosis based on histology, imaging, or clinical criteria; (3) availability of complete EGD and abdominal ultrasonography data within 7 days of each other during the same hospitalization. Exclusion criteria are detailed in Figure 1. Ultimately, 575 patients were enrolled and randomly divided into training (*n* = 403) and validation (*n* = 172) cohorts at a 7:3 ratio. Patients with incomplete clinical records were removed at this screening stage (*n* = 36, Figure 1); the 575 patients who entered the study had complete data for the outcome and for every candidate predictor.

**Figure 1:**
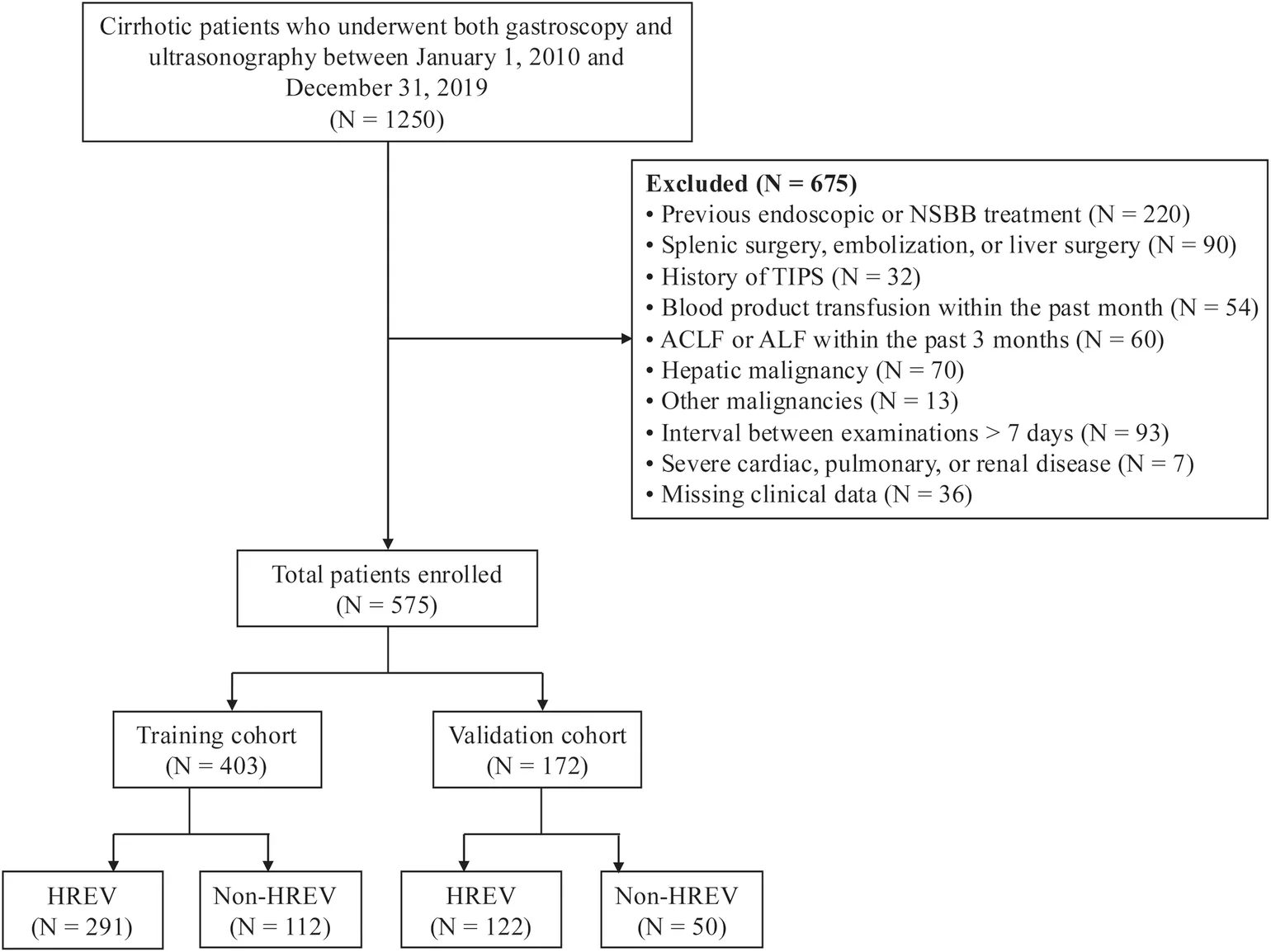
.Study flowchart. A total of 1,250 cirrhotic patients were initially screened. After applying the exclusion criteria, 575 eligible patients were enrolled and randomly divided into a training cohort (*n* = 403) and a validation cohort (*n* = 172). All participants underwent both endoscopy and abdominal ultrasonography. HREV was defined as medium-to-large varices (≥5 mm), varices of any size with red color signs, or small varices in patients with Child-Pugh class C.

All participants provided written informed consent. This study was approved by the Research Ethics Committee of the Third Affiliated Hospital of Sun Yat-sen University, and written informed consent was obtained from all participants. The ethics committee approval number is [2021]02-339.

### 2.2 Endoscopic evaluation

EGD was performed by trained endoscopists using Olympus GIF-HQ290 systems. Esophageal varices were graded according to the Japanese Research Society for Portal Hypertension guidelines. High-risk esophageal varices were defined, in accordance with the Baveno VII consensus, as medium-to-large varices (≥5 mm), varices of any size with red color signs, or small varices in patients with Child-Pugh class C.

### 2.3 Data collection

Demographic, clinical, and laboratory data were extracted from electronic medical records. Laboratory tests included complete blood count, liver and renal function, and coagulation profiles. Abdominal ultrasonography was performed by two independent experienced sonographers using a standardized protocol (MyLab ClassC Advanced, Esaote, Italy). Endoscopists and sonographers were blinded to each other's findings to prevent assessment bias. The following parameters were measured: left liver diameter (LLD), left liver thickness (LLT), right liver thickness (RLT), portal vein diameter (PVD), spleen diameter (SD), spleen thickness (SPT), and splenic vein diameter (SVD).

Examinations were performed after an overnight fast of at least 8 h with a 3.5–5.0 MHz convex transducer. For the three parameters retained in the final model, measurements were standardized as follows. RLT was measured with the patient supine during quiet respiration, on a sagittal section through the right mid-clavicular line, as the maximal anteroposterior thickness of the right lobe perpendicular to the liver capsule. PVD was measured at end-expiration, in the hepatoduodenal ligament approximately 1–2 cm distal to the formation of the main portal vein and proximal to its bifurcation, on a plane perpendicular to the vessel axis and from inner edge to inner edge. SD was measured with the patient in the right lateral decubitus position, as the maximal longitudinal (bipolar) diameter on a left intercostal coronal section displaying the splenic hilum. Each parameter was measured twice and the mean value was recorded; formal inter-observer reliability testing was not performed.

### 2.4 Statistical analysis

Statistical analyses were conducted using R software (version 4.4.3). Continuous variables are presented as median [interquartile range] and were compared using the Mann–Whitney U test; categorical variables are presented as frequencies (percentages) and were compared using the chi-square test with continuity correction. All 575 patients had complete data for the outcome and for all 29 candidate predictors.

Variables significant (*P* < 0.05) in univariate logistic regression were entered into a multivariable logistic regression model, and those that remained significant (*P* < 0.05) were retained as independent predictors. Multicollinearity among the retained predictors was assessed using the variance inflation factor (VIF), with VIF < 5 considered acceptable. Because such strategies may yield unstable selection and optimistic coefficients, two additional analyses were performed: LASSO logistic regression with 10-fold cross-validation, reported at *λ*min and *λ*1se (Supplementary Table S4, Supplementary Figure S4); and repetition of the entire selection procedure in 1,000 bootstrap resamples, from which the selection frequency of each variable and the proportion of resamples preserving the direction and statistical significance of each coefficient were derived (Supplementary Tables S2, S3). Linearity in the log odds was examined for every continuous predictor using restricted cubic splines with three knots, comparing the spline and linear models by likelihood-ratio test (Supplementary Table S2, Supplementary Figure S3).

A nomogram was constructed using the “rms” package in R software. Model performance was evaluated in terms of discrimination, calibration, and clinical utility. Discrimination was quantified by the area under the receiver operating characteristic curve (AUROC). Calibration was assessed using calibration curves together with the calibration intercept, calibration slope, and Brier score. Internal validation additionally used 1,000 bootstrap resamples to estimate the optimism-corrected C-index. The probability threshold maximizing the Youden index was derived in the training cohort, fixed, and then applied unchanged to the validation cohort; the threshold maximizing the Youden index within the validation cohort itself is reported for description only and is not presented as an independently validated clinical threshold (Supplementary Table S5). Because endoscopic capacity rather than a statistical criterion determines triage in practice, the thresholds corresponding to endoscoping the highest-risk 20% to 80% of patients, together with the resulting sensitivity, positive predictive value and number of HREV missed, are tabulated separately (Supplementary Table S8).

AUROCs were compared using the DeLong test. The incremental predictive value of the nomogram relative to the PC/SD ratio and FIB-4 was assessed by the categorical net reclassification improvement (NRI) and the integrated discrimination improvement (IDI), with 95% confidence intervals obtained from 1,000 bootstrap resamples. Three predicted-risk categories were defined *a priori* for the categorical NRI, low (<20%), intermediate (20%–80%) and high (≥80%), corresponding to non-expedited evaluation, genuine clinical uncertainty, and expedited endoscopy; these categories were not derived from the data and were applied identically to both cohorts and to both comparators (Supplementary Table S6). The nomogram was additionally compared with each of its own components measured in the same patients, with spleen thickness and splenic vein diameter, and with two reduced models (Supplementary Table S7, Supplementary Figure S2). Clinical net benefit was evaluated by decision-curve analysis (DCA) across a range of threshold probabilities. The clinical action modelled in the DCA was referral for EGD rather than initiation of prophylaxis, so the threshold probability represents the individual risk of HREV at which a clinician would consider endoscopic evaluation warranted; given the cohort prevalence of 71.8%, thresholds below approximately 0.50 imply endoscoping virtually all patients, and net benefit is therefore reported over the informative range of 0.50–0.90 (Supplementary Table S10).

The nomogram was compared with four established non-invasive scores (PC/SD ratio, FIB-4, APRI, and AAR) and with a simple elastography-free rule (platelet count > 150 × 10⁹/L and spleen diameter < 140 mm), which substitutes spleen size for liver stiffness in the spirit of the Baveno VI rule-out strategy (7, 20), as an elastography-free rule-out comparator. A subgroup analysis restricted to HBV-related cirrhosis, and a recalibration of the model intercept to lower expected prevalences, were performed *post hoc* (Supplementary Tables S12 and S14). A two-tailed *P* < 0.05 was considered statistically significant.

## 3 Results

### 3.1 Patient characteristics

A total of 575 patients with cirrhosis admitted to our institution met the inclusion criteria and were enrolled in the study (Figure 1). Stepwise comparisons of baseline characteristics between the HREV group (*n* = 413) and the non-HREV group (*n* = 162) are detailed in Table 1. The prevalence of HREV in the total cohort was 71.8%. Patients in the HREV group were significantly more likely to be male (79.2% vs. 69.8%; *P* = 0.022), had a higher prevalence of ascites (*P* < 0.001), and exhibited worse liver function and coagulation profiles (lower ALB, PTA; higher INR, TB). Regarding ultrasonographic parameters, the HREV group showed significantly lower RLT but higher LLT, PVD and SD (all *P* ≤ 0.010).

**Table 1:** Baseline characteristics of patients with HREV and non-HREV.

Baseline characteristics	Total (*N* = 575)	Non-HREV (*N* = 162)	HREV (*N* = 413)	*P* value
Age (year)	51.00 [44.00, 60.00]	52.00 [45.00, 59.00]	51.00 [44.00, 60.00]	0.947
Male (n, %)	440 (76.5)	113 (69.8)	327 (79.2)	0.022
Etiology, HBV (n, %)	385 (67.0)	101 (62.3)	284 (68.8)	0.170
Ascites (n, %)	306 (53.2)	50 (30.9)	256 (62.0)	<0.001
Hepatic encephalopathy (n, %)	19 (3.3)	2 (1.2)	17 (4.1)	0.139
Spontaneous peritonitis (n, %)	53 (9.2)	12 (7.4)	41 (9.9)	0.436
Laboratory test				
WBC (10⁹/L)	4.25 [3.22, 5.80]	4.82 [3.83, 6.38]	4.15 [3.00, 5.44]	<0.001
RBC (10¹²/L)	3.65 [3.03, 4.25]	4.11 [3.37, 4.55]	3.47 [2.95, 4.07]	<0.001
HGB (g/L)	114.00 [89.00, 131.00]	126.00 [110.00, 139.00]	105.00 [85.00, 126.00]	<0.001
PLT (10⁹/L)	78.00 [56.00, 110.00]	98.50 [73.50, 152.50]	71.00 [52.00, 98.00]	<0.001
NEUT (10⁹/L)	2.47 [1.73, 3.64]	2.61 [1.94, 3.82]	2.39 [1.69, 3.57]	0.041
LYMPH (10⁹/L)	1.13 [0.76, 1.48]	1.42 [1.08, 1.88]	1.00 [0.68, 1.34]	<0.001
NLR	2.35 [1.58, 3.52]	1.83 [1.29, 2.94]	2.53 [1.79, 3.68]	<0.001
AST (U/L)	54.00 [36.00, 82.50]	55.00 [31.50, 91.50]	53.00 [38.00, 79.00]	0.959
ALT (U/L)	40.00 [26.00, 68.50]	40.00 [27.00, 74.75]	39.00 [25.00, 66.00]	0.370
ALB (g/L)	33.30 [29.25, 38.15]	35.25 [29.92, 41.08]	32.90 [28.90, 37.20]	<0.001
TB (μmol/L)	26.00 [15.05, 47.20]	21.00 [11.85, 38.45]	26.50 [16.70, 49.60]	<0.001
DB (μmol/L)	12.00 [5.80, 24.45]	10.20 [4.40, 20.73]	12.70 [6.50, 25.10]	0.003
ALP (U/L)	102.00 [74.00, 136.00]	102.00 [74.00, 133.50]	102.00 [74.00, 137.00]	0.933
BUN (mmol/L)	4.83 [3.82, 6.06]	4.67 [3.74, 5.71]	4.87 [3.89, 6.29]	0.068
CREAT (μmol/L)	72.00 [61.00, 84.00]	72.00 [61.00, 80.00]	72.00 [62.00, 85.00]	0.329
PT (s)	16.50 [15.10, 18.50]	15.30 [14.20, 17.18]	16.90 [15.50, 19.00]	<0.001
PTA (%)	64.00 [53.00, 76.00]	74.00 [60.25, 86.00]	62.00 [51.00, 73.00]	<0.001
INR	1.33 [1.19, 1.54]	1.21 [1.10, 1.41]	1.37 [1.23, 1.60]	<0.001
APTT (s)	42.00 [38.70, 46.40]	41.90 [37.80, 46.00]	42.10 [39.10, 46.50]	0.187
Ultrasound				
LLD (mm)	64.00 [50.00, 79.00]	66.00 [50.00, 81.75]	63.00 [50.00, 77.00]	0.473
LLT (mm)	60.00 [54.00, 69.00]	59.00 [52.25, 65.00]	61.00 [55.00, 70.00]	0.010
RLT (mm)	99.00 [90.00, 106.00]	101.50 [96.00, 108.00]	97.00 [90.00, 105.00]	<0.001
PVD (mm)	12.00 [11.00, 13.00]	12.00 [11.00, 12.00]	12.00 [11.00, 13.00]	<0.001
SPT (mm)	47.00 [40.00, 55.00]	40.00 [35.00, 48.00]	50.00 [43.00, 57.00]	<0.001
SD (mm)	137.00 [117.00, 160.00]	117.00 [102.50, 137.00]	145.00 [126.00, 164.00]	<0.001
SVD (mm)	8.00 [6.00, 10.00]	6.00 [6.00, 8.00]	9.00 [7.00, 10.00]	<0.001
Score				
Child-Pugh Score	8.00 [6.00, 9.50]	6.00 [5.00, 8.00]	8.00 [7.00, 10.00]	<0.001
Child-Pugh Class A (n, %)	182 (31.7)	82 (50.6)	100 (24.2)	<0.001
Child-Pugh Class B (n, %)	249 (43.3)	58 (35.8)	191 (46.2)	0.029
Child-Pugh Class C (n, %)	144 (25.0)	22 (13.6)	122 (29.5)	<0.001
APRI	1.84 [1.02, 3.14]	1.41 [0.72, 2.83]	1.96 [1.19, 3.33]	<0.001
FIB-4	5.74 [3.40, 9.04]	4.17 [2.41, 7.29]	6.21 [3.95, 9.32]	<0.001
PC/SD	0.57 [0.37, 0.88]	0.86 [0.57, 1.35]	0.50 [0.34, 0.72]	<0.001
AAR	1.30 [1.00, 1.72]	1.21 [0.95, 1.63]	1.31 [1.01, 1.75]	0.093
EGD				
Red Sign (n, %)	275 (47.8)	0 (0.0)	275 (66.6)	<0.001
Diameter of EVs (mm)	8.00 [5.00, 10.00]	0.00 [0.00, 4.00]	10.00 [8.00, 10.00]	<0.001

Data are presented as median [interquartile range] for continuous variables and *n* (%) for categorical variables. *P* values were calculated using the Mann–Whitney U test for continuous variables and the chi-square test with continuity correction for categorical variables. A *P* value <0.05 was considered statistically significant.

HREV, high-risk esophageal varices; WBC, white blood cell count; RBC, red blood cell count; HGB, hemoglobin; PLT, platelet count; NEUT, neutrophil count; LYMPH, lymphocyte count; NLR, neutrophil-to-lymphocyte ratio; AST, aspartate aminotransferase; ALT, alanine aminotransferase; ALB, albumin; TB, total bilirubin; DB, direct bilirubin; ALP, alkaline phosphatase; BUN, blood urea nitrogen; CREAT, creatinine; PT, prothrombin time; PTA, prothrombin time activity; INR, international normalized ratio; APTT, activated partial thromboplastin time; LLD, left liver diameter; LLT, left liver thickness; RLT, right liver thickness; PVD, portal vein diameter; SPT, spleen thickness; SD, spleen diameter; SVD, splenic vein diameter; APRI, AST to platelet ratio index; FIB-4, fibrosis-4 index; PC/SD, platelet count to spleen diameter ratio; AAR, AST/ALT ratio; EGD, esophagogastroduodenoscopy; EVs, esophageal varices.

The cohort was randomly divided into a training set (*n* = 403) and a validation set (*n* = 172). As shown in Table 2, there were no statistically significant differences in demographic, clinical, laboratory, or ultrasonographic characteristics between the two cohorts (no reported *P* value was <0.05), indicating a balanced distribution of baseline variables.

**Table 2:** Summary of study variables grouped by training and validation cohort.

Baseline characteristics	Training Cohort (*N* = 403)	Validation Cohort (*N* = 172)	*P* value
HREV (n, %)	291 (72.2)	122 (70.9)	0.833
Age (year)	52.00 [45.00, 60.00]	49.00 [44.00, 59.00]	0.050
Male (n, %)	308 (76.4)	132 (76.7)	1.000
Etiology, HBV (n, %)	266 (66.0)	119 (69.2)	0.518
Laboratory test			
WBC (10⁹/L)	4.31 [3.22, 5.78]	4.19 [3.17, 5.89]	0.381
RBC (10¹²/L)	3.63 [3.02, 4.24]	3.68 [3.06, 4.28]	0.553
HGB (g/L)	113.00 [91.00, 130.00]	117.50 [85.00, 132.00]	0.885
PLT (10⁹/L)	77.00 [56.00, 110.50]	78.00 [57.00, 106.25]	0.968
NEUT (10⁹/L)	2.47 [1.73, 3.74]	2.46 [1.74, 3.57]	0.488
LYMPH (10⁹/L)	1.11 [0.76, 1.45]	1.15 [0.74, 1.56]	0.786
NLR	2.40 [1.62, 3.56]	2.32 [1.46, 3.35]	0.372
AST (U/L)	54.00 [37.00, 84.50]	53.00 [35.75, 77.50]	0.624
ALT (U/L)	40.00 [26.50, 68.00]	39.00 [25.00, 68.25]	0.698
ALB (g/L)	33.10 [29.20, 37.80]	34.65 [29.30, 39.38]	0.173
TB (μmol/L)	26.30 [15.25, 47.75]	24.60 [14.50, 46.02]	0.818
DB (μmol/L)	12.25 [6.06, 24.45]	11.40 [5.47, 24.45]	0.470
ALP (U/L)	102.00 [74.00, 137.50]	102.00 [72.75, 132.25]	0.514
BUN (mmol/L)	4.83 [3.79, 6.10]	4.83 [3.89, 5.87]	0.955
CREAT (μmol/L)	72.00 [62.00, 84.00]	71.00 [61.00, 82.25]	0.498
PT (s)	16.40 [15.10, 18.40]	16.60 [15.17, 18.80]	0.256
PTA (%)	65.00 [54.00, 76.00]	63.00 [51.75, 75.00]	0.301
INR	1.32 [1.18, 1.52]	1.37 [1.20, 1.58]	0.229
APTT (s)	42.00 [38.55, 46.40]	41.95 [39.10, 46.42]	0.804
Ultrasound			
LLD (mm)	64.00 [50.00, 80.00]	63.50 [50.00, 76.00]	0.682
LLT (mm)	61.00 [54.00, 69.50]	61.00 [54.75, 68.00]	0.780
RLT (mm)	100.00 [90.00, 106.00]	99.00 [91.00, 106.00]	0.641
PVD (mm)	12.00 [11.00, 13.00]	12.00 [11.00, 13.00]	0.729
SPT (mm)	47.00 [41.00, 55.00]	47.00 [39.00, 55.00]	0.373
SD (mm)	137.00 [118.00, 160.50]	137.00 [114.00, 154.50]	0.298
SVD (mm)	8.00 [6.00, 10.00]	7.00 [6.00, 10.00]	0.285
Score			
Child-Pugh Score	8.00 [6.00, 9.00]	8.00 [6.00, 10.00]	0.910
Child-Pugh Class A (n, %)	129 (32.0)	53 (30.8)	0.854
Child-Pugh Class B (n, %)	178 (44.2)	71 (41.3)	0.583
Child-Pugh Class C (n, %)	96 (23.8)	48 (27.9)	0.352
APRI	1.83 [1.03, 3.14]	1.84 [0.99, 3.14]	0.762
FIB-4	5.74 [3.28, 9.07]	5.74 [3.57, 8.76]	0.793
PC/SD	0.57 [0.37, 0.87]	0.59 [0.38, 0.91]	0.703
AAR	1.30 [0.99, 1.73]	1.29 [1.05, 1.70]	0.659
EGD			
Red Sign (n, %)	200 (49.6)	75 (43.6)	0.218
Diameter of EVs (mm)	10.00 [5.00, 10.00]	8.00 [5.00, 10.00]	0.088

Data are presented as median [interquartile range] for continuous variables and *n* (%) for categorical variables. Differences between the training and validation cohorts were assessed using the Mann–Whitney U test for continuous variables and the chi-square test with continuity correction for categorical variables. A *P* value <0.05 was considered statistically significant.

WBC, white blood cell count; RBC, red blood cell count; HGB, hemoglobin; PLT, platelet count; NEUT, neutrophil count; LYMPH, lymphocyte count; NLR, neutrophil-to-lymphocyte ratio; AST, aspartate aminotransferase; ALT, alanine aminotransferase; ALB, albumin; TB, total bilirubin; DB, direct bilirubin; ALP, alkaline phosphatase; BUN, blood urea nitrogen; CREAT, creatinine; PT, prothrombin time; PTA, prothrombin time activity; INR, international normalized ratio; APTT, activated partial thromboplastin time; LLD, left liver diameter; LLT, left liver thickness; RLT, right liver thickness; PVD, portal vein diameter; SPT, spleen thickness; SD, spleen diameter; SVD, splenic vein diameter; APRI, AST to platelet ratio index; FIB-4, fibrosis-4 index; PC/SD, platelet count to spleen diameter ratio; AAR, AST/ALT ratio.

### 3.2 Identification of predictors for high-risk esophageal varices

In the training cohort, univariate logistic regression identified 15 variables significantly associated with HREV (Table 3), including hematological indices, coagulation markers, and ultrasonographic parameters. These candidate variables were entered into a multivariable logistic regression analysis. Ultimately, multivariate analysis identified six independent predictors representing distinct pathophysiological aspects of portal hypertension: male sex (OR 2.06, 95% CI 1.118–3.803), HGB and PLT (markers of hypersplenism/systemic disease), alongside morphological parameters RLT, PVD, and SD. Variance Inflation Factor (VIF) analysis suggested that despite biological correlations, no prohibitive multicollinearity existed among these variables (all VIF < 5; range 1.05–1.32). Odds ratios expressed per clinically meaningful increment, which are easier to interpret than per-unit estimates for variables such as HGB and SD, are given in Supplementary Table S13.

**Table 3:** Univariate and multivariate analysis of variables according to HREV in the training cohort.

Variables	Univariate analysis			Multivariate analysis		
	OR	95% CI	*P* value	OR	95% CI	*P* value
Male sex	1.98	1.217–3.227	0.006	2.06	1.118–3.803	0.020
WBC	0.92	0.860–0.986	0.018			
RBC	0.57	0.440–0.743	<0.001			
HGB	0.97	0.966–0.984	<0.001	0.98	0.971–0.992	<0.001
PLT	0.99	0.983–0.992	<0.001	0.99	0.987–0.998	0.010
LYMPH	0.46	0.325–0.658	<0.001			
ALB	0.96	0.926–0.992	0.016			
PT	1.14	1.050–1.235	0.002			
PTA	0.97	0.962–0.986	<0.001			
INR	3.25	1.507–7.002	0.003			
RLT	0.98	0.959–0.993	0.005	0.96	0.943–0.984	<0.001
PVD	1.49	1.278–1.737	<0.001	1.38	1.157–1.651	<0.001
SPT	1.09	1.059–1.115	<0.001			
SD	1.03	1.023–1.042	<0.001	1.02	1.005–1.028	0.005
SVD	1.45	1.288–1.634	<0.001			

Univariate and multivariate logistic regression analyses were performed to identify predictors of high-risk esophageal varices. Variables demonstrating statistical significance (*P* < 0.05) in the univariate analysis were subsequently included in the multivariate logistic regression model. Odds ratios and 95% confidence intervals are Wald-based estimates; the final model was refitted with the six retained predictors, whose full coefficients are given in Supplementary Table S1.

OR, odds ratio; CI, confidence interval; WBC, white blood cell count; RBC, red blood cell count; HGB, hemoglobin; PLT, platelet count; LYMPH, lymphocyte count; ALB, albumin; PT, prothrombin time; PTA, prothrombin time activity; INR, international normalized ratio; RLT, right liver thickness; PVD, portal vein diameter; SPT, spleen thickness; SD, spleen diameter; SVD, splenic vein diameter.

Kendall's tau-b correlation analysis (Figure 2) further corroborated the biological plausibility of these predictors. PVD and SD showed a positive correlation with EV grade (r = 0.21 and 0.33, respectively; *P* < 0.001), reflecting portal hypertension severity. Conversely, HGB, PLT, and RLT exhibited negative correlations with EV grade, consistent with hypersplenism and liver parenchymal atrophy in advanced cirrhosis.

**Figure 2:**
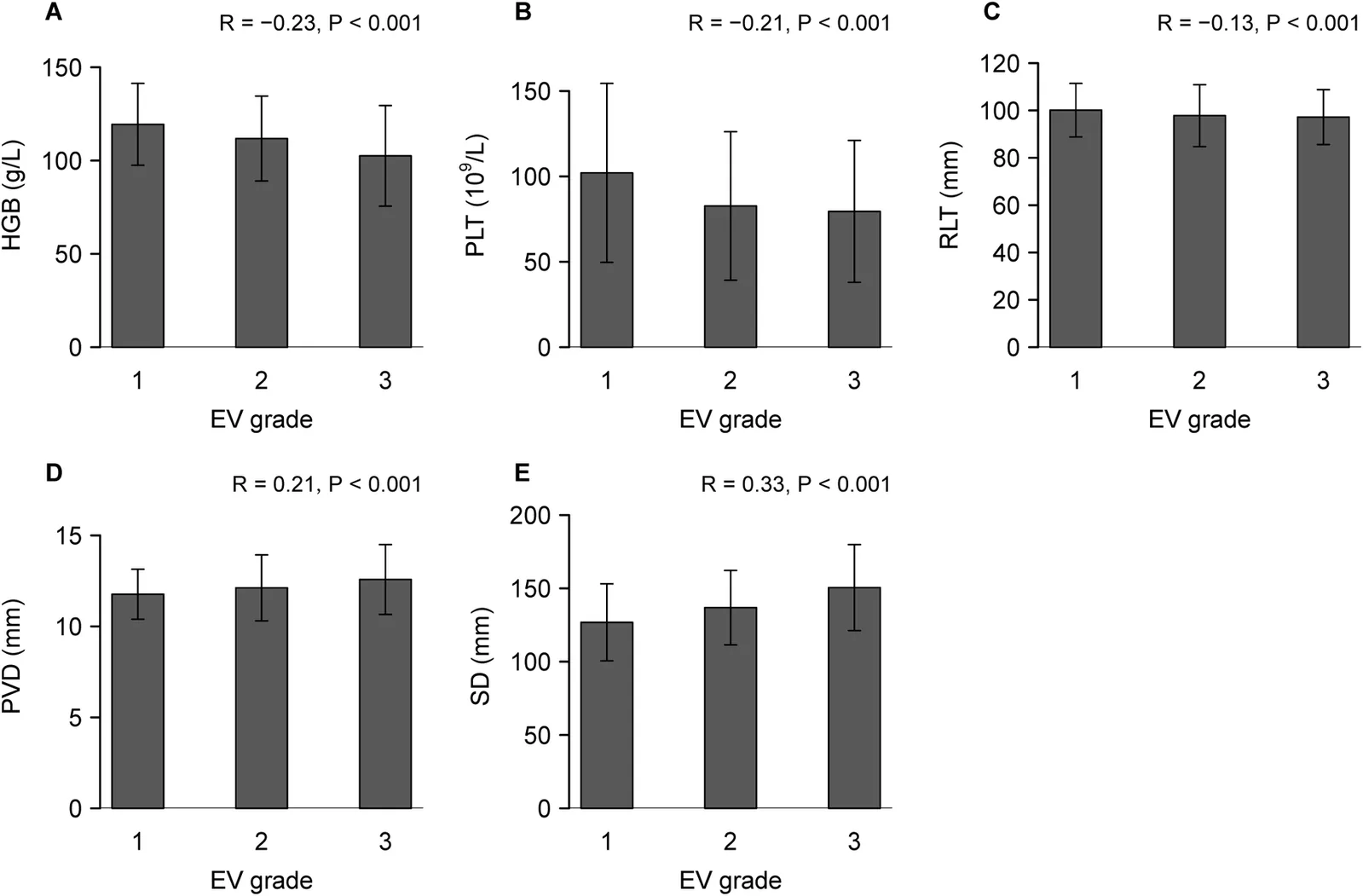
Correlations between independent predictors and esophageal varices grades. **(A)** Hemoglobin (HGB); **(B)** platelet count (PLT); **(C)** right liver thickness (RLT); **(D)** portal vein diameter (PVD); and **(E)** spleen diameter (SD) in relation to the grading of esophageal varices (EV). PVD and SD were positively correlated with EV severity (Kendall's tau-b r = 0.21 and 0.33, respectively; *P* < 0.001), while HGB, PLT, and RLT exhibited negative correlations (r = −0.23, −0.21, and −0.13, respectively; *P* < 0.001). EV1, mild; EV2, moderate; EV3, severe.

### 3.3 Nomogram construction

A nomogram (HREV-nomogram) was constructed based on the six identified independent predictors (Figure 3). The HREV-nomogram demonstrated satisfactory discrimination, achieving an AUROC of 0.803 (95% CI: 0.755–0.850) in the training cohort and 0.807 (95% CI: 0.734–0.880) in the validation cohort (Figures 4A,D).

**Figure 3:**
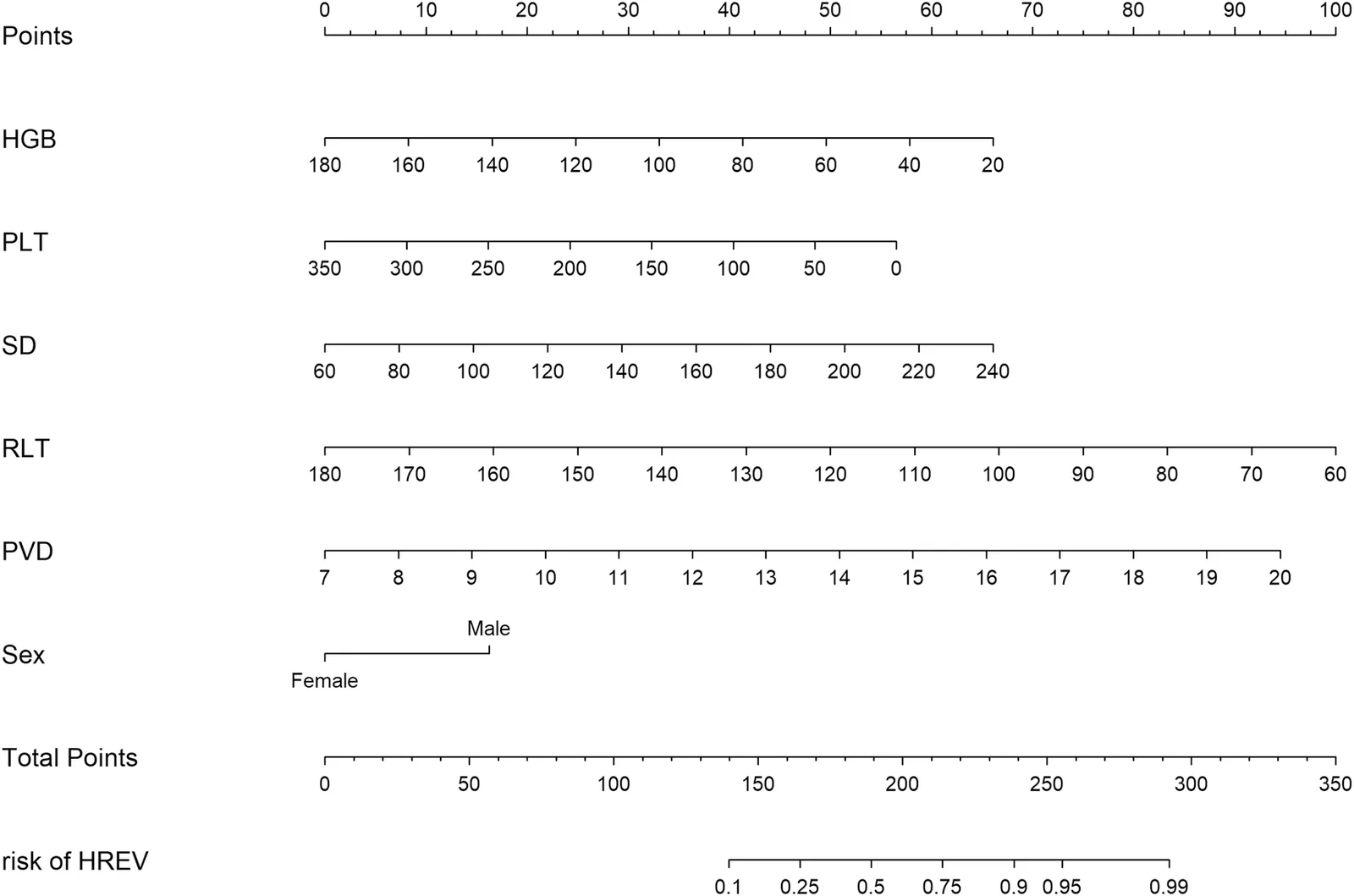
Nomogram for predicting the probability of high-risk esophageal varices (HREV). The nomogram integrates six routinely available parameters: male sex, hemoglobin (HGB), platelet count (PLT), right liver thickness (RLT), portal vein diameter (PVD), and spleen diameter (SD). To use the nomogram, locate the patient's value for each variable on the corresponding axis, draw a line upward to the “Points” axis to determine the score, sum the scores for all variables to obtain the “Total Points”, and draw a vertical line downward to estimate the risk probability.

**Figure 4:**
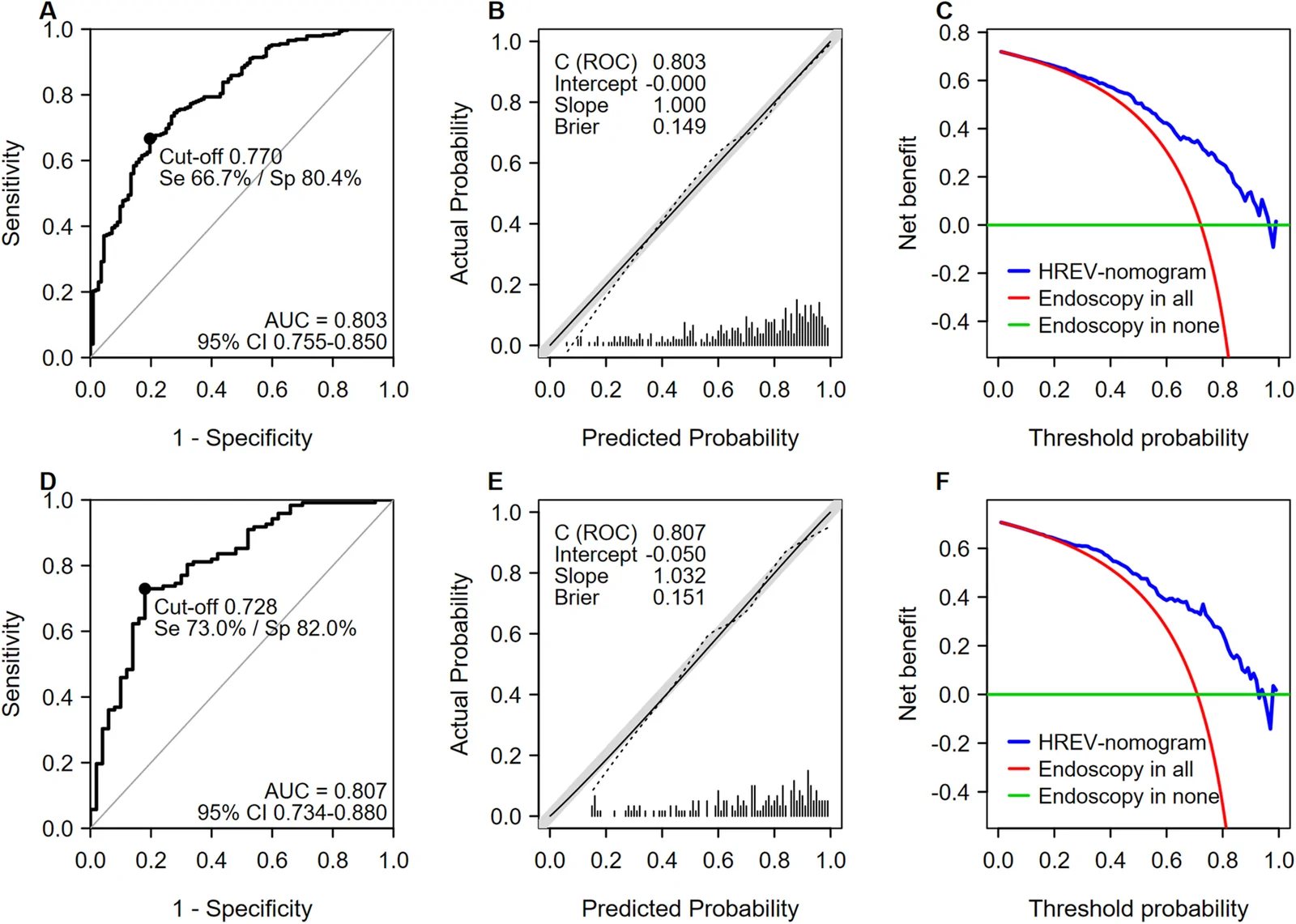
Performance of the HREV-nomogram in the training and validation cohorts. **(A,D)** Receiver operating characteristic (ROC) curves in the training **(A)** and validation **(D)** cohorts. Panel **A** shows the training-cohort Youden threshold of 0.770 (sensitivity 66.7%, specificity 80.4%). Panel **D** shows the validation-cohort-specific Youden threshold of 0.728 (sensitivity 73.0%, specificity 82.0%), which is presented for descriptive purposes only. **(B,E)** Calibration curves comparing predicted (x-axis) and observed (y-axis) probabilities in the training **(B)** and validation **(E)** cohorts; the diagonal line represents ideal prediction. **(C,F)** Decision curve analysis (DCA) in the training **(C)** and validation **(F)** cohorts. The blue line represents the HREV-nomogram, while the red and green lines represent “Endoscopy in all” and “Endoscopy in none”, respectively.

The maximum Youden index in the training cohort corresponded to a threshold of 0.770 (sensitivity 66.7%, specificity 80.4%). This training-derived threshold was fixed and applied unchanged to the validation cohort, where it yielded a sensitivity of 63.9% (95% CI 55.1–71.9), a specificity of 82.0% (69.2–90.2), a positive predictive value of 89.7% (81.5–94.5) and a negative predictive value of 48.2% (37.9–58.7), classifying 50.6% of patients as high risk; approximately half of the patients classified as low risk nevertheless had HREV (51.8%). The threshold maximizing the Youden index within the validation cohort itself (0.728) is reported for description only (Supplementary Table S5). Across the three predefined risk categories, the observed prevalence of HREV rose from 0.0% (training) and 12.5% (validation) in the low-risk band (<20%) to 90.6% and 91.0% in the high-risk band (≥80%); only 3.0% and 4.7% of patients fell into the low-risk band (Supplementary Table S6).

Calibration curves for both cohorts showed excellent agreement between the predicted and observed probabilities of HREV (Figures 4B,E). Furthermore, decision curve analysis (DCA) demonstrated that the HREV-nomogram provided a superior clinical net benefit compared to the “treat-all” or “treat-none” strategies across a wide range of threshold probabilities (Figures 4C,F); over the informative threshold range of 0.50–0.90 the net benefit of the nomogram exceeded that of endoscoping all patients, corresponding at a threshold of 0.70 to approximately 11 (training) and 13 (validation) endoscopies avoided per 100 patients without loss of HREV detected (Supplementary Table S10). Quantitatively, the calibration intercept and slope were 0.000 and 1.000 in the training cohort and −0.050 and 1.032 in the validation cohort (ideal values of 0 and 1), with Brier scores of 0.149 and 0.151, respectively; internal validation using 1,000 bootstrap resamples yielded an optimism-corrected C-index of 0.788, indicating minimal overfitting (Table 4).

**Table 4:** Discrimination and calibration of the HREV-nomogram in the training and validation cohorts.

Metric	Training cohort (*n* = 403)	Validation cohort (*n* = 172)
C-index (AUROC)	0.803 (0.755–0.850)	0.807 (0.734–0.880)
Optimism-corrected C-index	0.788	—
Calibration intercept	0.000 (−0.252–0.252)	−0.050 (−0.406–0.353)
Calibration slope	1.000 (0.768–1.232)	1.032 (0.669–1.396)
Brier score	0.149	0.151

Values in parentheses are 95% confidence intervals. The optimism-corrected C-index was estimated from 1,000 bootstrap resamples of the training cohort and is not reported for the validation cohort. The calibration intercept and slope have ideal values of 0 and 1, respectively, and lower Brier scores indicate better predictive accuracy.

AUROC, area under the receiver operating characteristic curve; CI, confidence interval.

### 3.4 Performance comparison of the HREV-nomogram with existing clinical models

We compared the diagnostic performance of the HREV-nomogram with established non-invasive models (APRI, AAR, FIB-4, and PC/SD; Table 5A, Figure 5A; the corresponding curves in the validation cohort are shown in Supplementary Figure S1). In the training cohort, the HREV-nomogram yielded the highest AUROC (0.803), significantly higher than PC/SD (0.720), FIB-4 (0.606), APRI (0.571), and AAR (0.524) (DeLong *P* < 0.001 for all four comparisons). In the validation cohort (AUROC 0.807), the HREV-nomogram remained significantly superior to AAR (0.594; DeLong *P* = 0.001) and APRI (0.695; *P* = 0.03), whereas its discrimination did not differ significantly from FIB-4 (0.737; *P* = 0.16) or the PC/SD ratio (0.763; *P* = 0.19). We further compared reclassification performance against FIB-4 and the PC/SD ratio. Relative to the PC/SD ratio, the categorical net reclassification improvement (NRI) was 0.199 (95% CI 0.091–0.308; *P* < 0.001) and the integrated discrimination improvement (IDI) was 0.116 (95% CI 0.080–0.153; *P* < 0.001) in the training cohort, and 0.207 (95% CI 0.036–0.377; *P* = 0.017) and 0.087 (95% CI 0.037–0.136; *P* < 0.001), respectively, in the validation cohort. Relative to FIB-4, the NRI was 0.502 (95% CI 0.373–0.632; *P* < 0.001) and the IDI 0.237 (95% CI 0.190–0.283; *P* < 0.001) in the training cohort, and 0.495 (95% CI 0.302–0.689; *P* < 0.001) and 0.223 (95% CI 0.153–0.292; *P* < 0.001), respectively, in the validation cohort. HREV-nomogram reclassified patients significantly better than both the FIB-4 index and the PC/SD ratio.

**Table 5:** Comparison of the HREV-nomogram with established non-invasive methods for predicting high-risk esophageal varices.

(A) Discrimination				
Method	Training AUROC (95% CI)	Validation AUROC (95% CI)	DeLong *P* vs. nomogram (Training)	DeLong *P* vs. nomogram (Validation)
Nomogram	0.803 (0.755–0.850)	0.807 (0.734–0.880)	Reference	Reference
PC/SD ratio	0.720 (0.663–0.777)	0.763 (0.683–0.844)	<0.001	0.19
FIB-4	0.606 (0.542–0.671)	0.737 (0.649–0.824)	<0.001	0.16
APRI	0.571 (0.505–0.638)	0.695 (0.601–0.790)	<0.001	0.03
AAR	0.524 (0.461–0.587)	0.594 (0.500–0.688)	<0.001	0.001

The discrimination of the HREV-nomogram was compared with four established non-invasive scores (PC/SD ratio, FIB-4, APRI, and AAR); the areas under the receiver operating characteristic curve were compared using the DeLong test, and 95% confidence intervals were estimated by the same method. A comparison with each individual predictor measured in the same patients is provided in Supplementary Table S7.

**Table:** 

(B) Diagnostic performance of a simple elastography-free rule as a rule-out rule		
Metric	Training cohort	Validation cohort
Endoscopies spared, *n* (%)	43 (10.7)	20 (11.6)
Sensitivity, % (95% CI)	94.8 (91.7–96.9)	94.3 (88.6–97.2)
Specificity, % (95% CI)	25.0 (17.9–33.8)	26.0 (15.9–39.6)
Positive predictive value, % (95% CI)	76.7 (72.0–80.7)	75.7 (68.3–81.8)
Negative predictive value, % (95% CI)	65.1 (50.2–77.6)	65.0 (43.3–81.9)
HREV missed among spared, %	34.9	35.0

The rule evaluated is a platelet count > 150 × 10⁹/L combined with a spleen diameter < 140 mm. “Endoscopies spared” denotes patients classified as low-risk and eligible to defer endoscopy; “HREV missed among spared” is the proportion of these low-risk patients who in fact had high-risk esophageal varices (equal to one minus the negative predictive value). The rule yields a single binary classification and therefore has no area under the receiver operating characteristic curve; its discrimination relative to the HREV-nomogram (DeLong test) is reported in the main text. Values in parentheses are 95% confidence intervals.

CI, confidence interval; HREV, high-risk esophageal varices.

**Table:** 

(C) Reclassification of the HREV-nomogram relative to the PC/SD ratio and FIB-4					
Comparator	Cohort	NRI (95% CI)	*P*	IDI (95% CI)	*P*
PC/SD ratio	Training	0.199 (0.091–0.308)	<0.001	0.116 (0.080–0.153)	<0.001
PC/SD ratio	Validation	0.207 (0.036–0.377)	0.017	0.087 (0.037–0.136)	<0.001
FIB-4	Training	0.502 (0.373–0.632)	<0.001	0.237 (0.190–0.283)	<0.001
FIB-4	Validation	0.495 (0.302–0.689)	<0.001	0.223 (0.153–0.292)	<0.001

Categorical net reclassification improvement (NRI) was calculated using three predefined risk categories (<20%, 20%–80%, ≥80%) applied identically to both cohorts and to both comparators (Supplementary Table S6); 95% confidence intervals were obtained from 1,000 bootstrap resamples. IDI, integrated discrimination improvement.

**Figure 5:**
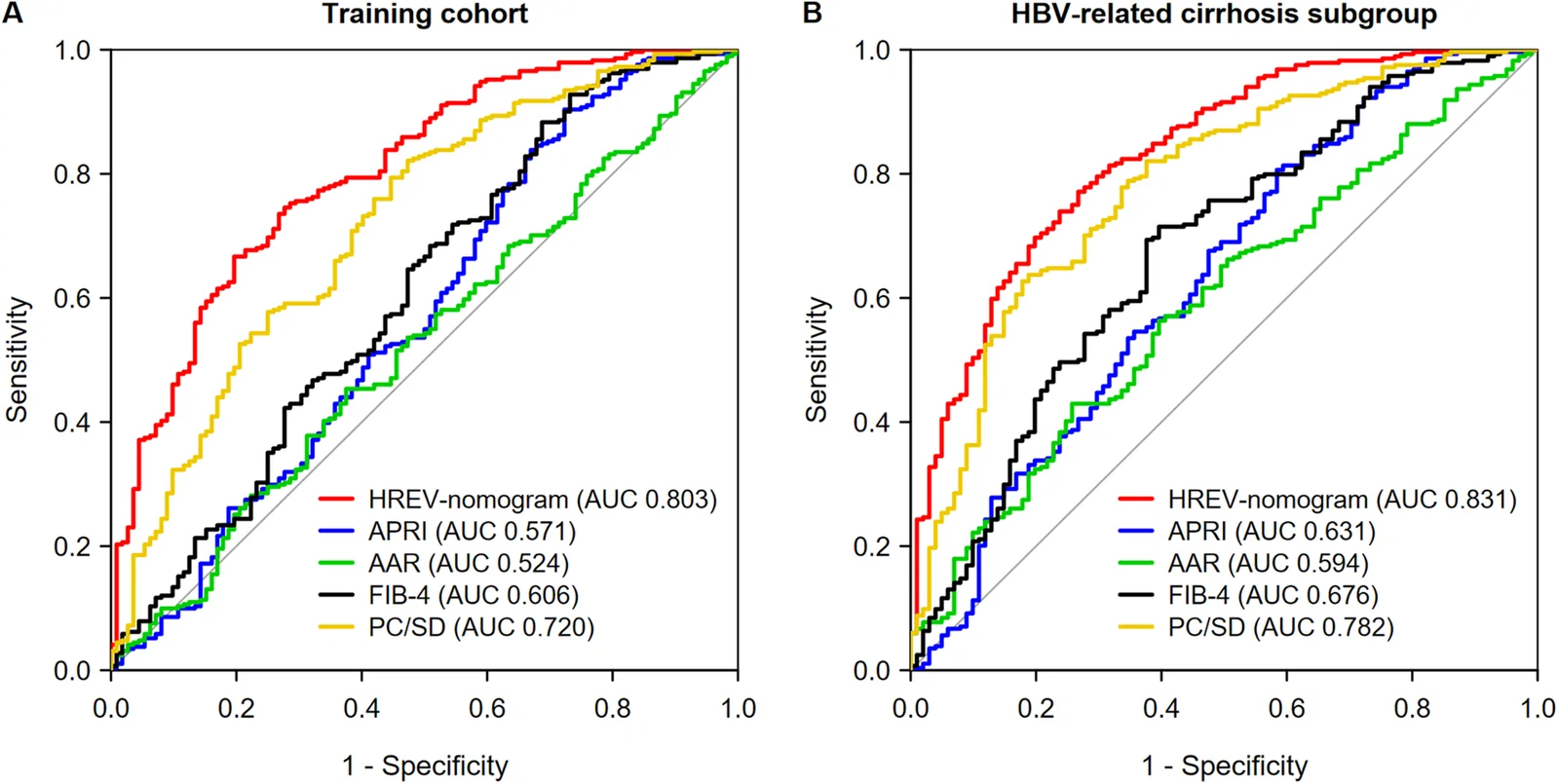
Comparison of the HREV-nomogram with established non-invasive models. **(A)** ROC curves comparing the HREV-nomogram with PC/SD, FIB-4, APRI, and AAR in the training cohort. **(B)** Comparison of diagnostic performance in the subgroup of patients with HBV-related cirrhosis. The nomogram was applied with the training-derived coefficients, without refitting, to all 385 patients with HBV-related cirrhosis (284 with HREV) and retained superior discrimination (AUROC 0.831, 95% CI 0.784-0.877).

Because APRI, AAR and FIB-4 were designed for fibrosis staging and contain no hemodynamic component, we also compared the nomogram with each of its own components measured in the same patients (Supplementary Table S7, Supplementary Figure S2). In the training cohort the six-variable model discriminated significantly better than every single measurement, including spleen diameter (0.803 vs. 0.742; *P* = 0.003), spleen thickness (0.733; *P* = 0.002), splenic vein diameter (0.738; *P* = 0.008), platelet count (0.678; *P* < 0.001) and hemoglobin (0.679; *P* < 0.001). In the validation cohort the corresponding advantage over spleen diameter alone (0.741) and platelet count alone (0.738) did not reach statistical significance (*P* = 0.061 and *P* = 0.053). A reduced four-variable model (HGB, PLT, SD and PVD) performed similarly to the full model (0.789 and 0.780; *P* = 0.263 and *P* = 0.100), indicating that the incremental contribution of RLT and male sex is modest.

We additionally assessed the simple elastography-free rule (platelet count > 150 × 10⁹/L and spleen diameter < 140 mm), the principal elastography-free rule-out rule (Table 5B): these criteria spared only 10.7% (training) and 11.6% (validation) of endoscopies while leaving 34.9% and 35.0% of HREV undetected among patients classified as low-risk (negative predictive value approximately 65%), well above the 5% missed-varices threshold generally considered acceptable for safely deferring endoscopy, and the HREV-nomogram discriminated significantly better than these criteria in both cohorts (DeLong *P* < 0.001 and *P* = 0.001). When the nomogram was constrained to spare exactly the same number of endoscopies (10.7% and 11.6%), it missed 9 of 43 and 3 of 20 HREV (20.9% and 15.0%) compared with 15 and 7 (34.9% and 35.0%) for the simple rule; both remain far above the 5% standard (Supplementary Table S9).

In the subgroup of patients with HBV-related cirrhosis, the HREV-nomogram retained higher discrimination than the other non-invasive models (Figure 5B). This *post hoc* analysis included all 385 patients with HBV-related cirrhosis (284 with HREV, 73.8%) and applied the training-derived coefficients without refitting; the AUROC was 0.831 (95% CI 0.784–0.877), significantly higher than the PC/SD ratio (0.782; *P* = 0.011), FIB-4 (0.676; *P* < 0.001), APRI (0.631; *P* < 0.001) and AAR (0.594; *P* < 0.001). Corresponding values were 0.844 (0.792–0.896) in the HBV training subgroup (*n* = 266, 199 events), 0.805 (0.712–0.898) in the HBV validation subgroup (*n* = 119, 85 events; not significantly different from the PC/SD ratio, *P* = 0.863) and 0.746 (0.671–0.821) in patients with non-HBV aetiologies (Supplementary Table S12).

### 3.5 Threshold behaviour under endoscopy-capacity constraints

Because the Youden index weights false-negative and false-positive classifications equally, whereas the practical constraint on triage is endoscopic capacity, we tabulated the consequences of endoscoping the highest-risk 20% to 80% of patients ranked by predicted probability (Supplementary Table S8). Endoscoping the highest-risk 20% (threshold 0.920) identified HREV in 95.1% and 94.3% of those scoped in the training and validation cohorts, at a sensitivity of 26.5% and 27.0%. Endoscoping the highest-risk 50% (thresholds 0.786 and 0.777) detected 62.2% and 63.9% of all HREV with a positive predictive value of 89.6% and 90.7%. Even at 80% capacity, 27 and 11 HREV remained undetected, indicating that capacity-based triage reorders rather than removes the need for endoscopy in a population of this risk profile.

### 3.6 Predictor stability, functional form and sensitivity analyses

All six predictors were retained by LASSO regression at *λ*1se, and the cross-validated LASSO models did not improve discrimination in the validation cohort (AUROC 0.792 at *λ*min and 0.794 at *λ*1se, versus 0.807 for the six-variable model; Supplementary Table S4, Supplementary Figure S4). Across 1,000 bootstrap resamples the direction of every coefficient was preserved in at least 99.0% of resamples, and the direction with *P* < 0.05 in 61.9% (male sex) to 96.7% (PVD). When the whole selection procedure was repeated within each resample, individual selection frequencies ranged from 20.3% (SD) to 91.7% (PVD); at least one spleen-size measurement (SD, SPT or SVD) was selected in 56.3% of resamples and at least one liver-morphology measurement (RLT, LLT or LLD) in 76.9%, indicating that the underlying constructs were reproduced more consistently than the specific proxy variables (Supplementary Tables S2, S3). Tests for non-linearity using restricted cubic splines were negative for HGB (*P* = 0.125), PLT (*P* = 0.538), RLT (*P* = 0.248) and PVD (*P* = 0.939) but positive for SD (*P* = 0.002); modelling SD with a spline changed discrimination only marginally (training 0.803–0.810; validation 0.807–0.796), so the linear term was retained (Supplementary Table S2, Supplementary Figure S3). Restricting the outcome to medium-to-large varices, by excluding or by reclassifying the 21 patients defined solely by the Child-Pugh C clause, yielded AUROCs of 0.805 and 0.790 in the training cohort and 0.819 and 0.806 in the validation cohort, with all odds ratios materially unchanged (Supplementary Table S15).

## 4 Discussion

In this study, we successfully developed and validated a novel, non-invasive nomogram for the prediction of high-risk esophageal varices (HREV) in patients with cirrhosis. The HREV-nomogram, integrating six routinely available parameters: male sex, hemoglobin (HGB), platelet count (PLT), right liver thickness (RLT), portal vein diameter (PVD), and spleen diameter (SD), demonstrated satisfactory diagnostic performance. It outperformed the established biochemical scores APRI, AAR and FIB-4 and improved risk reclassification over the platelet count-to-spleen diameter (PC/SD) ratio, while showing good calibration and clinical utility (19, 21). Rather than replacing current gold standards, this tool addresses a critical diagnostic gap in resource-limited settings. It offers a means of ranking patients for endoscopic evaluation where advanced elastography is unavailable, so that endoscopic capacity and prophylactic intervention can be directed first to those at highest risk (6, 13).

The progression of portal hypertension to variceal hemorrhage represents a lethal turning point in the natural history of cirrhosis (2, 3, 22). Current guidelines strictly advocate for primary prophylaxis (NSBBs or band ligation) once HREV is identified (5, 7, 8). While esophagogastroduodenoscopy (EGD) and hepatic venous pressure gradient (HVPG) measurement remain the gold standards for diagnosis and risk stratification, their widespread implementation is hindered by invasiveness, cost, and technical complexity, particularly in resource-constrained settings (10–12). To mitigate the need for invasive screening, the Baveno VI criteria (LSM <20 kPa and PLT >150 × 10⁹/L) have been widely adopted to rule out HREV (7), concepts that were reaffirmed and refined in the recent Baveno VII consensus (14). However, the reliance on Transient Elastography (TE) limits its applicability in primary care centers lacking elastography equipment. Furthermore, TE accuracy can be compromised by obesity, ascites, or narrow intercostal spaces (15, 16). Consequently, while the Baveno criteria remain the reference standard for non-invasively ruling out varices, a diagnostic gap persists in resource-constrained settings. Our model addresses this specific unmet need by offering a viable alternative that leverages solely standard abdominal ultrasonography and routine blood tests—resources that are routine even in grassroots medical institutions.

Notably, even the simple elastography-free rule that substitutes spleen diameter for liver stiffness (20), performed poorly in our cohort: they would have spared only 11% of endoscopies while missing 35% of HREV (negative predictive value approximately 65%), far above the missed-varices rate considered acceptable for a safe rule-out rule. The nomogram was better but not adequate for this purpose: at the same sparing rate it still missed 20.9% and 15.0% of HREV. This indicates that simple elastography-free thresholds are insufficient in a high-prevalence population such as ours and supports the need for an integrated, population-adapted model such as the HREV-nomogram. Performance of the rule was strongly dependent on liver function: in Child-Pugh A patients (prevalence 54.9%) it spared 15.9% of endoscopies with 6.9% of HREV missed (negative predictive value 93.1%), whereas in Child-Pugh B/C patients it missed 58.8%, and the nomogram itself discriminated better in Child-Pugh A than in Child-Pugh B/C patients (AUROC 0.825 vs. 0.767; Supplementary Table S11, Supplementary Figure S5), used for prioritisation rather than exclusion.

The variables constituting the HREV-nomogram are not merely statistical associations but reflect the integrated pathophysiology of advanced cirrhosis and portal hypertension. Male sex was identified as an independent risk factor, consistent with epidemiological data showing higher morbidity and mortality in men with chronic liver disease, potentially linked to lifestyle factors and higher HBV prevalence in this demographic (1, 23). The inverse correlation we observed between these markers and variceal severity aligns with previous findings that hemoglobin (HGB) and platelet count (PLT) are surrogate markers of the systemic consequences of portal hypertension (24, 25). Anemia in cirrhosis is multifactorial, resulting from chronic gastrointestinal blood loss, nutritional deficiencies, bone marrow suppression, and hemodilution due to plasma volume expansion (26). Thrombocytopenia is primarily driven by portal hypertensive splenomegaly with consequent platelet sequestration and destruction, compounded by reduced hepatic thrombopoietin synthesis (27).

The ultrasonographic parameters provide direct anatomical insights. Right Liver Thickness (RLT) reduction reflects the parenchymal atrophy and architectural distortion characteristic of advanced cirrhosis, particularly the “right lobe atrophy/left lobe hypertrophy” phenomenon (28). Conversely, Portal Vein Diameter (PVD) and Spleen Diameter (SD) serve as direct sonographic indicators of elevated portal pressure and congestive splenomegaly (29, 30). Our data confirmed that PVD and SD are positively correlated with variceal grade, reinforcing their hemodynamic relevance. The integration of these serological and morphological markers into a single model provides a more comprehensive risk assessment than any single parameter, although the size of that gain depends on the sample: the six-variable model discriminated significantly better than spleen diameter alone in the training cohort (0.803 vs. 0.742; *P* = 0.003) but not in the smaller validation cohort (0.807 vs. 0.741; *P* = 0.061). Whether a six-variable instrument is worth its added complexity relative to spleen diameter alone therefore remains to be settled in an independent sample, and we regard this as a primary question for external validation.

A key strength of this study is the superior discriminatory power of the HREV-nomogram compared to traditional biochemical scores. The nomogram achieved AUROCs exceeding 0.80 in both training and validation cohorts, significantly outperforming the biochemical scores APRI and AAR. Although its AUROC advantage over FIB-4 and the PC/SD ratio did not reach statistical significance in the validation cohort, the HREV-nomogram significantly improved net reclassification (NRI and IDI) over both comparators in both cohorts (19, 21). While scores like FIB-4 are excellent for fibrosis staging, they lack the specific hemodynamic components (like spleen size or portal vein width) necessary to accurately reflect portal pressure. Notably, our model maintained high accuracy within the HBV-related cirrhosis subgroup (AUROC 0.831 with fixed coefficients), which is of paramount importance given that HBV remains the predominant etiology of liver disease in China and many Asian regions (4, 23).

The choice of a decision threshold deserves comment. The Youden index gives equal weight to a missed HREV and to an unnecessary endoscopy, which does not reflect the clinical reality of triage, where a missed varix carries the risk of haemorrhage while an unnecessary endoscopy consumes a scarce appointment. In practice the operating point is set by capacity. Our capacity table shows the trade-off explicitly: endoscoping the highest-risk fifth of patients yields a positive predictive value above 94% but detects barely a quarter of HREV, whereas endoscoping the highest-risk 80% detects more than 90%. We therefore present the Youden-derived threshold as a descriptive reference and recommend that implementing centres select the operating point from the capacity table rather than from a statistical criterion.

The visualization of our model as a nomogram maximizes its practical utility, enabling clinicians to rapidly estimate individual risk at the bedside. The decision curve analysis (DCA) further confirmed that using the HREV-nomogram to guide risk stratification decisions provides a greater net benefit than “treat-all” or “treat-none” strategies across a wide range of threshold probabilities (31); because the action modelled is referral for endoscopy rather than treatment, the informative range of threshold probabilities in a cohort of this prevalence is 0.50–0.90. This risk-stratification capability is primarily suited to prioritizing high-risk individuals for prompt evaluation and prophylactic intervention. Given the high HREV prevalence in this referral cohort and the limited rule-out performance of simple non-invasive thresholds discussed above, using the nomogram to defer endoscopy in apparently low-risk patients would require confirmation in lower-prevalence, prospectively and externally validated settings before routine adoption. For centres wishing to explore transportability, Supplementary Table S14 provides the intercept adjustment required to recalibrate the model to expected local prevalences between 10% and 60% with the six slope coefficients held fixed.

Crucially, our model possesses potential clinical utility for deployment in primary healthcare institutions in China. Given the marked disparities in economic development and healthcare resource allocation across regions, especially in underdeveloped areas like Northwest China, many primary care facilities lack access to advanced cross-sectional imaging modalities such as CT or MRI (32). While AI-assisted diagnostic technologies have seen rapid progress, they frequently necessitate high-quality imaging data or complex biomarkers. Hampered by hardware constraints, these sophisticated tools face significant barriers to implementation in primary care settings. In stark contrast, our model integrates only routine laboratory parameters and standard abdominal ultrasonography, resources that are ubiquitously available in the vast majority of grassroots hospitals. By bridging the gap between technological innovation and clinical feasibility, this model serves as a cost-effective, accessible risk stratification tool for resource-constrained regions, ultimately promoting greater equity in healthcare delivery (33).

Significantly, the HREV-nomogram is well-suited for implementation within the “tiered diagnosis and treatment system,” a strategy vital for optimizing healthcare delivery in China, particularly in remote and resource-limited regions. We propose a clinical pathway where physicians in primary care settings utilize this nomogram for initial risk assessment. Patients identified as high-risk would be prioritized for immediate referral to superior medical centers (tertiary hospitals) for confirmatory EGD and potential prophylactic intervention, whereas patients with lower predicted risk would continue to be managed according to existing guideline-based surveillance rather than having endoscopy withheld: with a negative predictive value below 50% at the operating threshold, a low predicted risk in this setting is a statement about queue position, not a license to omit endoscopy. This workflow not only aligns with China's national conditions by mitigating the shortage of specialists in grassroots hospitals but also ensures that critical endoscopic resources are precisely targeted toward the patients who benefit most.

Despite its strengths, our study has several limitations that must be acknowledged. First, its retrospective, single-center design may introduce selection bias. The study population was drawn from a tertiary hospital, which may overrepresent patients with more advanced or decompensated disease, as evidenced by the high prevalence of HREV (71.8%). This may limit the generalizability of the specific probability estimates to community-based or early-stage cirrhotic populations, although the model's strong discriminatory ability is encouraging. Future multicenter prospective studies are needed to recalibrate the model for community-based populations. Second, while the model performed well in an HBV-predominant cohort, its generalizability to other etiologies (e.g., NAFLD, alcohol-associated cirrhosis) requires external validation; discrimination was already lower in the non-HBV subgroup (AUROC 0.746). Then, we did not directly compare our model with spleen stiffness measurements (SSM) or TE, as these were not routinely performed in all patients during the study period; however, since LSM is not routinely performed in many primary care centers, the target setting for our model, we focused on developing a tool independent of elastography to ensure broader applicability. Additionally, the model underwent internal validation only (a random training–validation split with bootstrap optimism correction); no independent external cohort was available, so the absolute probability estimates require external validation before clinical use. The reproducibility of the ultrasonographic measurements was not formally assessed (e.g., inter-observer agreement or intraclass correlation coefficients), although all examinations followed a standardized protocol performed by experienced sonographers blinded to endoscopic findings; formal inter-observer testing is a prerequisite for multicentre implementation, since three of the six predictors are operator-dependent.

Finally, predictors were selected by univariable screening followed by retention of statistically significant variables. This strategy is widely used but is known to produce unstable selection and coefficients biased away from the null, and the optimism-corrected C-index of 0.788 quantifies only part of that optimism. Our stability analyses were reassuring for the direction of every coefficient but showed that individual variables, particularly spleen diameter, were selected inconsistently when the entire procedure was repeated in bootstrap resamples, reflecting collinearity among spleen measurements. Spleen diameter was retained because it is reported routinely and used by existing non-invasive rules, but a different proxy for splenic congestion would probably perform similarly. In addition, spleen diameter showed statistical evidence of a non-linear association with the log odds of HREV; a linear term was retained deliberately so that the nomogram remains readable by hand, at a small cost in fit.

## 5 Conclusions

In conclusion, we have developed and internally validated a nomogram for the non-invasive assessment of high-risk esophageal varices in cirrhotic patients. By synthesizing easily obtainable clinical and ultrasonographic data, this tool offers a practical and inexpensive way to rank patients by risk in settings where transient elastography is unavailable, so that endoscopic capacity and prophylactic intervention can be directed first to those most likely to benefit. In a cohort with 71.8% prevalence its negative predictive value was insufficient to justify deferring endoscopy in patients classified as low risk, and the model should not be used for that purpose on current evidence. Prospective, multi-center studies in lower-prevalence populations, with recalibration and formal assessment of the reproducibility of the ultrasonographic measurements, are now warranted to establish whether the model can also support safe rule-out and to assess its impact on clinical decision-making and patient prognosis.

## Data Availability

The datasets generated and analyzed during the current study are available from the corresponding author on reasonable request.

## Glossary

AAR: AST/ALT ratio
ALB: albumin
ALP: alkaline phosphatase
ALT: alanine aminotransferase
APRI: AST to Platelet Ratio Index
APTT: activated partial thromboplastin time
AST: aspartate aminotransferase
AUROC: area under the receiver operating characteristic curve
BUN: blood urea nitrogen
CI: confidence interval
CREAT: creatinine
DB: direct bilirubin
DCA: decision-curve analysis
EGD: esophagogastroduodenoscopy
EVs: esophageal varices
FIB-4: Fibrosis-4 index
GOV: gastroesophageal varices/esophagogastric varices
HBV: hepatitis B virus
HGB: hemoglobin
HREV: high-risk esophageal varices
HVPG: hepatic venous pressure gradient
INR: international normalized ratio
LASSO: least absolute shrinkage and selection operator
LLD: left liver diameter
LLT: left liver thickness
LSM: liver stiffness measurement
LYMPH: lymphocyte count
NEUT: neutrophil count
NLR: neutrophil-to-lymphocyte ratio
NPV: negative predictive value
NSBBs: non-selective beta-blockers
OR: odds ratio
PC/SD: platelet count to spleen diameter ratio
PH: portal hypertension
PLT: platelet count
PPV: positive predictive value
PT: prothrombin time
PTA: prothrombin time activity
PVD: portal vein diameter
RBC: red blood cell count
RCS: restricted cubic spline
RLT: right liver thickness
ROC: receiver operating characteristic
SD: spleen diameter
SPT: spleen thickness
SVD: splenic vein diameter
TB: total bilirubin
TE: transient elastography
VIF: variance inflation factor
WBC: white blood cell count.
