# Supplementary material for: A non-invasive nomogram for high-risk esophageal varices in cirrhosis without transient elastography: development and internal validation
Source: Front Med (Lausanne). 2026 Sep 18;13:1946799. doi: 10.3389/fmed.2026.1946799 (PMC13630700; doi:10.3389/fmed.2026.1946799)
Supplement: Supplementary file 1 [file Datasheet1.docx]

**Supplementary Material**

**A Non-invasive Nomogram for High-Risk Esophageal Varices in Cirrhosis without Transient Elastography: Development and Internal Validation**

Yi Wu^1,2^, Junyang Luo^3^, Shuixian Yang^1^, Shuaishuai Kong^1^, Dan Dan Chen^1^, Wenhui Guo^1^, Nianfeng Fan^4^, Zebin Wu^1^, Yurong Gu^1^, Zhaoxia Hu^1^, Zhiliang Gao^1,2^, Yubao Zheng^1,2,#^

**Contents**

Table S1. Coefficients of the multivariable logistic regression model.

Table S2. Predictor-selection stability and functional form.

Table S3. Variable-selection frequency across 1000 bootstrap resamples.

Table S4. LASSO logistic regression coefficients.

Table S5. Diagnostic performance at the fixed training-derived threshold.

Table S6. Predefined predicted-risk categories used for the categorical NRI.

Table S7. Nomogram versus individual predictors and reduced models.

Table S8. Thresholds defined by endoscopy capacity.

Table S9. Comparison at a matched endoscopy-sparing rate.

Table S10. Net benefit at key threshold probabilities.

Table S11. Rule and model performance by Child-Pugh stratum.

Table S12. HBV subgroup analysis using fixed model coefficients.

Table S13. Odds ratios per clinically meaningful increment.

Table S14. Intercept recalibration for lower-prevalence settings.

Table S15. Sensitivity analysis of the outcome definition.

Figure S1. ROC curves of all models in the validation cohort.

Figure S2. ROC curves of individual predictors versus the full model (validation cohort).

Figure S3. Restricted cubic spline curves for the five continuous predictors.

Figure S4. LASSO cross-validation curve and coefficient paths.

Figure S5. ROC curves of the nomogram by Child-Pugh stratum.

**Supplementary Table S1. Coefficients of the multivariable logistic regression model underlying the HREV-nomogram (training cohort, n = 403).**

| **Variable (unit)** | **β** | **SE** | **OR** | **95% CI** | **P value** |
| --- | --- | --- | --- | --- | --- |
| Male sex (vs. female) | 0.724 | 0.312 | 2.06 | 1.118–3.803 | 0.020 |
| Hemoglobin, HGB (g/L) | −0.018 | 0.006 | 0.98 | 0.971–0.992 | <0.001 |
| Platelet count, PLT (×10⁹/L) | −0.007 | 0.003 | 0.99 | 0.987–0.998 | 0.010 |
| Right liver thickness, RLT (mm) | −0.037 | 0.011 | 0.96 | 0.943–0.984 | <0.001 |
| Portal vein diameter, PVD (mm) | 0.324 | 0.091 | 1.38 | 1.157–1.651 | <0.001 |
| Spleen diameter, SD (mm) | 0.016 | 0.006 | 1.02 | 1.005–1.028 | 0.005 |
| Intercept (β₀) | 0.838 | 1.506 | — | — | — |

β = log-odds regression coefficient; SE = standard error. β, SE and P values are the maximum-likelihood estimates from the fitted multivariable logistic model. Odds ratios (OR) and 95% confidence intervals are as reported in Table 3. Reference category for sex is female; continuous predictors were modelled on their original scale (units in parentheses).

**Supplementary Table S2. Stability of predictor selection and assessment of functional form for the six variables of the HREV-nomogram.**

| **Predictor** | **Bootstrap resamples with same coefficient direction, %** | **Bootstrap resamples with same direction and P < 0.05, %** | **Retained by LASSO at λ1se** | **Non-linearity LR test, P** | **AUROC with spline term, training** | **AUROC with spline term, validation** |
| --- | --- | --- | --- | --- | --- | --- |
| HGB | 100.0 | 91.0 | Yes | 0.125 | 0.803 | 0.796 |
| PLT | 99.5 | 74.4 | Yes | 0.538 | 0.802 | 0.803 |
| SD | 99.8 | 81.0 | Yes | 0.002 | 0.810 | 0.796 |
| RLT | 99.7 | 87.5 | Yes | 0.248 | 0.807 | 0.799 |
| PVD | 100.0 | 96.7 | Yes | 0.939 | 0.803 | 0.808 |
| Male sex | 99.0 | 61.9 | Yes | Not applicable | — | — |

Bootstrap analysis used 1000 resamples of the training cohort with the final six-variable model refitted in each resample. LASSO used 10-fold cross-validation; λ1se additionally retained LYMPH, SPT and SVD. Linearity in the log odds was tested by comparing a restricted cubic spline (3 knots) with the linear term by likelihood-ratio test; reference AUROCs of the fully linear model are 0.803 (training) and 0.807 (validation). Spleen diameter showed evidence of non-linearity, but the spline model did not improve validation discrimination, so the linear term was retained.

**Supplementary Table S3. Frequency with which each candidate variable was selected when the entire selection procedure was repeated in 1000 bootstrap resamples of the training cohort.**

| **Variable** | **Selection frequency, %** | **Variable** | **Selection frequency, %** |
| --- | --- | --- | --- |
| PVD | 91.7 | RLT | 69.2 |
| Male sex | 56.0 | PLT | 49.7 |
| SVD | 38.3 | HGB | 36.0 |
| Etiology, HBV | 24.5 | SD | 20.3 |
| LLD | 16.1 | RBC | 12.8 |
| AST | 10.9 | NLR | 9.2 |
| LLT | 7.4 | LYMPH | 5.4 |
| SPT | 5.0 | APTT | 2.4 |
| Age | 2.2 | BUN | 2.2 |
| ALP | 1.9 | CREAT | 1.8 |
| WBC | 1.7 | ALT | 1.2 |
| NEUT | 0.8 |  |  |

In each bootstrap resample, univariable screening (P < 0.05) followed by multivariable retention (P < 0.05) was repeated from the start. At least one spleen-size measurement (SD, SPT or SVD) was selected in 56.3% of resamples and at least one liver-morphology measurement (RLT, LLT or LLD) in 76.9%, indicating that the underlying constructs were reproduced more consistently than any individual proxy. Variables never selected are omitted.

**Supplementary Table S4. LASSO logistic regression coefficients in the training cohort.**

| **Variable** | **β at λmin** | **Standardised β at λmin** | **β at λ1se** | **Retained at λ1se** | **In final nomogram** |
| --- | --- | --- | --- | --- | --- |
| PVD | 0.26145 | 0.473 | 0.12914 | Yes | Yes |
| HGB | −0.01373 | −0.365 | −0.00949 | Yes | Yes |
| RLT | −0.02489 | −0.320 | −0.00795 | Yes | Yes |
| SVD | 0.11007 | 0.313 | 0.06872 | Yes | No |
| SD | 0.01003 | 0.304 | 0.00845 | Yes | Yes |
| PLT | −0.00556 | −0.289 | −0.00386 | Yes | Yes |
| Male sex | 0.59352 | 0.252 | 0.06625 | Yes | Yes |
| Etiology, HBV | 0.35055 | 0.166 | 0 | No | No |
| APTT | −0.02300 | −0.154 | 0 | No | No |
| RBC | −0.17249 | −0.151 | 0 | No | No |
| ALP | 0.00179 | 0.106 | 0 | No | No |
| Age | 0.00817 | 0.096 | 0 | No | No |
| NLR | 0.03210 | 0.088 | 0 | No | No |
| LLD | −0.00335 | −0.076 | 0 | No | No |
| CREAT | −0.00232 | −0.070 | 0 | No | No |
| AST | −0.00099 | −0.060 | 0 | No | No |
| LLT | 0.00247 | 0.029 | 0 | No | No |
| LYMPH | −0.03555 | −0.027 | −0.02394 | Yes | No |
| SPT | 0 | 0 | 0.00005 | Yes | No |
| WBC, NEUT, ALT, BUN | 0 | 0 | 0 | No | No |

Ten-fold cross-validated LASSO logistic regression on the 23 candidate variables that are not components of the Child-Pugh score. Validation-cohort AUROC was 0.792 at λmin and 0.794 at λ1se, compared with 0.807 for the six-variable HREV-nomogram, i.e. penalised regression did not improve discrimination. Standardised β is the coefficient multiplied by the standard deviation of the variable.

**Supplementary Table S5. Diagnostic performance of the HREV-nomogram at the fixed training-derived probability threshold.**

| **Metric** | **Training (n = 403), ≥0.770** | **Validation (n = 172), ≥0.770 (fixed)** | **Validation (n = 172), ≥0.728 (descriptive)** |
| --- | --- | --- | --- |
| Sensitivity, % (95% CI) | 66.7 (61.1–71.8) | 63.9 (55.1–71.9) | 73.0 (64.5–80.0) |
| Specificity, % (95% CI) | 80.4 (72.0–86.7) | 82.0 (69.2–90.2) | 82.0 (69.2–90.2) |
| Positive predictive value, % (95% CI) | 89.8 (85.1–93.2) | 89.7 (81.5–94.5) | 90.8 (83.5–95.1) |
| Negative predictive value, % (95% CI) | 48.1 (41.1–55.3) | 48.2 (37.9–58.7) | 55.4 (44.1–66.2) |
| Classified high risk, n (%) | 216 (53.6) | 87 (50.6) | 98 (57.0) |
| Classified low risk, n (%) | 187 (46.4) | 85 (49.4) | 74 (43.0) |
| HREV among patients classified low risk, % | 51.9 | 51.8 | 44.6 |
| Overall accuracy, % | 70.5 | 69.2 | 75.6 |

The threshold of 0.770 was derived in the training cohort by the maximum Youden index and applied unchanged to the validation cohort. The third column reports the threshold that maximised the Youden index within the validation cohort itself and is descriptive only; it is not an independently validated threshold. Roughly half of the patients classified as low risk had HREV at either threshold, which is why the model is not proposed as a rule-out instrument.

**Supplementary Table S6. Predefined predicted-risk categories used for the categorical net reclassification improvement.**

| **Predicted-risk category** | **Training, n (%)** | **HREV within category, n (%)** | **Validation, n (%)** | **HREV within category, n (%)** |
| --- | --- | --- | --- | --- |
| Low (<20%) | 12 (3.0) | 0 (0.0) | 8 (4.7) | 1 (12.5) |
| Intermediate (20–80%) | 199 (49.4) | 117 (58.8) | 86 (50.0) | 50 (58.1) |
| High (≥80%) | 192 (47.6) | 174 (90.6) | 78 (45.3) | 71 (91.0) |

Categories were defined a priori on clinical grounds, non-expedited evaluation, genuine clinical uncertainty, and expedited endoscopy, were not derived from the data, and were applied identically to the training and validation cohorts and to both comparators (FIB-4 and PC/SD ratio). Percentages in the second and fourth columns are of the cohort; percentages in the third and fifth columns are of patients within that category.

**Supplementary Table S7. Discrimination of the HREV-nomogram compared with each individual predictor and with reduced models.**

| **Predictor or model** | **Training AUROC (95% CI)** | **DeLong P vs nomogram** | **Validation AUROC (95% CI)** | **DeLong P vs nomogram** |
| --- | --- | --- | --- | --- |
| Full HREV-nomogram (6 variables) | 0.803 (0.755–0.850) | Reference | 0.807 (0.734–0.880) | Reference |
| Spleen diameter (SD) | 0.742 (0.686–0.799) | 0.003 | 0.741 (0.660–0.822) | 0.061 |
| Platelet count (PLT) | 0.678 (0.619–0.737) | <0.001 | 0.738 (0.657–0.819) | 0.053 |
| Hemoglobin (HGB) | 0.679 (0.620–0.738) | <0.001 | 0.723 (0.640–0.806) | 0.039 |
| Right liver thickness (RLT) | 0.594 (0.532–0.656) | <0.001 | 0.673 (0.586–0.760) | 0.010 |
| Portal vein diameter (PVD) | 0.667 (0.610–0.723) | <0.001 | 0.575 (0.485–0.664) | <0.001 |
| Spleen thickness (SPT) | 0.733 (0.676–0.790) | 0.002 | 0.714 (0.630–0.798) | 0.015 |
| Splenic vein diameter (SVD) | 0.738 (0.683–0.793) | 0.008 | 0.692 (0.608–0.776) | 0.002 |
| PC/SD ratio | 0.720 (0.663–0.777) | <0.001 | 0.763 (0.683–0.844) | 0.190 |
| Reduced model: PLT + SD + PVD + RLT | 0.776 (0.724–0.827) | 0.017 | 0.758 (0.677–0.839) | 0.017 |
| Reduced model: HGB + PLT + SD + PVD | 0.789 (0.736–0.841) | 0.263 | 0.780 (0.704–0.856) | 0.100 |

All comparisons are paired within the same patients and were tested with the DeLong test. Reduced models were refitted in the training cohort and applied unchanged to the validation cohort. The advantage of the full model over spleen diameter alone and platelet count alone was significant in the training cohort but not in the smaller validation cohort.

**Supplementary Table S8. Operating characteristics when the highest-risk proportion of patients is selected for endoscopy (capacity-based thresholds).**

| **Cohort** | **Endoscopy capacity** | **Threshold** | **Patients scoped, n** | **HREV detected, n** | **Sensitivity, %** | **PPV, %** | **HREV missed, n** |
| --- | --- | --- | --- | --- | --- | --- | --- |
| Training | Top 20% by risk | 0.920 | 81 | 77 | 26.5 | 95.1 | 214 |
| Training | Top 30% by risk | 0.888 | 121 | 113 | 38.8 | 93.4 | 178 |
| Training | Top 40% by risk | 0.843 | 162 | 147 | 50.5 | 90.7 | 144 |
| Training | Top 50% by risk | 0.786 | 202 | 181 | 62.2 | 89.6 | 110 |
| Training | Top 60% by risk | 0.721 | 242 | 212 | 72.9 | 87.6 | 79 |
| Training | Top 70% by risk | 0.639 | 283 | 234 | 80.4 | 82.7 | 57 |
| Training | Top 80% by risk | 0.516 | 323 | 264 | 90.7 | 81.7 | 27 |
| Validation | Top 20% by risk | 0.920 | 35 | 33 | 27.0 | 94.3 | 89 |
| Validation | Top 30% by risk | 0.876 | 52 | 47 | 38.5 | 90.4 | 75 |
| Validation | Top 40% by risk | 0.831 | 69 | 62 | 50.8 | 89.9 | 60 |
| Validation | Top 50% by risk | 0.777 | 86 | 78 | 63.9 | 90.7 | 44 |
| Validation | Top 60% by risk | 0.703 | 104 | 90 | 73.8 | 86.5 | 32 |
| Validation | Top 70% by risk | 0.625 | 121 | 100 | 82.0 | 82.6 | 22 |
| Validation | Top 80% by risk | 0.524 | 138 | 111 | 91.0 | 80.4 | 11 |

Patients were ranked by predicted probability and the stated proportion with the highest risk was assumed to undergo endoscopy. The threshold column gives the predicted probability at that capacity in each cohort. PPV, positive predictive value. This table is intended to replace a single statistically derived cut-off as the basis for implementation.

**Supplementary Table S9. Head-to-head comparison of the HREV-nomogram and the simple elastography-free rule at a matched endoscopy-sparing rate.**

| **Cohort** | **Endoscopies spared, n (%)** | **HREV missed by the simple rule, n (%)** | **HREV missed by the nomogram, n (%)** | **Nomogram threshold used** |
| --- | --- | --- | --- | --- |
| Training | 43 (10.7) | 15 (34.9) | 9 (20.9) | 0.364 |
| Validation | 20 (11.6) | 7 (35.0) | 3 (15.0) | 0.373 |

The nomogram threshold was set so that exactly the same number of endoscopies was spared as by the simple rule (platelet count > 150 × 10⁹/L and spleen diameter < 140 mm). Percentages of missed HREV are of the patients spared. Both instruments miss far more than the 5% generally accepted for deferring endoscopy.

**Supplementary Table S10. Net benefit of the HREV-nomogram at key threshold probabilities.**

| **Threshold probability** | **Nomogram, training** | **Endoscope all, training** | **Nomogram, validation** | **Endoscope all, validation** |
| --- | --- | --- | --- | --- |
| 0.50 | 0.504 | 0.444 | 0.488 | 0.419 |
| 0.60 | 0.423 | 0.305 | 0.387 | 0.273 |
| 0.70 | 0.342 | 0.074 | 0.339 | 0.031 |
| 0.80 | 0.253 | −0.390 | 0.250 | −0.453 |
| 0.90 | 0.136 | −1.779 | 0.064 | −1.907 |

The clinical action modelled is referral for endoscopy rather than initiation of treatment; the threshold probability is the risk of HREV at which endoscopic evaluation would be considered warranted. Given a cohort prevalence of 71.8%, thresholds below approximately 0.50 imply endoscoping nearly all patients, so the informative range is 0.50–0.90. At a threshold of 0.70 the difference in net benefit corresponds to approximately 11 (training) and 13 (validation) endoscopies avoided per 100 patients at an unchanged number of HREV detected.

**Supplementary Table S11. Performance of the simple elastography-free rule and of the HREV-nomogram by Child-Pugh stratum.**

| **Stratum** | **N** | **HREV, n** | **HREV prevalence, %** | **Endoscopies spared by rule, %** | **HREV missed by rule, %** | **Rule NPV, %** | **Nomogram AUROC (95% CI)** |
| --- | --- | --- | --- | --- | --- | --- | --- |
| Child-Pugh A | 182 | 100 | 54.9 | 15.9 | 6.9 | 93.1 | 0.825 (0.765–0.884) |
| Child-Pugh B/C | 393 | 313 | 79.6 | 8.7 | 58.8 | 41.2 | 0.767 (0.709–0.825) |
| All patients | 575 | 413 | 71.8 | 11.0 | 34.9 | 65.1 | 0.804 (0.764–0.843) |

Analysis of the whole study population with the training-derived coefficients. Rule-out performance of the simple rule is acceptable only in Child-Pugh A patients, illustrating the dependence of negative predictive value on prevalence and disease severity. NPV, negative predictive value.

**Supplementary Table S12. Discrimination of the HREV-nomogram in the HBV subgroup using the fixed training-derived coefficients.**

| **Subgroup** | **N** | **HREV, n (%)** | **Nomogram AUROC (95% CI)** | **PC/SD AUROC (P)** | **FIB-4 AUROC (P)** | **APRI AUROC (P)** | **AAR AUROC (P)** |
| --- | --- | --- | --- | --- | --- | --- | --- |
| HBV-related cirrhosis (all patients) | 385 | 284 (73.8) | 0.831 (0.784–0.877) | 0.782 (0.011) | 0.676 (<0.001) | 0.631 (<0.001) | 0.594 (<0.001) |
| HBV — training cohort | 266 | 199 (74.8) | 0.844 (0.792–0.896) | 0.767 (<0.001) | 0.634 (<0.001) | 0.578 (<0.001) | 0.577 (<0.001) |
| HBV — validation cohort | 119 | 85 (71.4) | 0.805 (0.712–0.898) | 0.811 (0.863) | 0.771 (0.546) | 0.744 (0.288) | 0.626 (0.023) |
| Non-HBV aetiologies | 190 | 129 (67.9) | 0.746 (0.671–0.821) | 0.632 (0.002) | 0.584 (0.002) | 0.567 (0.002) | 0.482 (<0.001) |

This subgroup analysis was post hoc and not prespecified. The nomogram was applied with the coefficients estimated in the training cohort, without refitting within the subgroup; P values are DeLong tests against the nomogram. Refitting the model within the HBV subgroup gives an apparent AUROC of 0.847, which is not comparable with the non-refitted comparator scores and is therefore not reported in the main text.

**Supplementary Table S13. Odds ratios expressed per clinically meaningful increment.**

| **Predictor** | **Increment** | **OR per increment (95% CI)** | **OR per unit as reported in Table 3** |
| --- | --- | --- | --- |
| HGB | per 10 g/L | 0.83 (0.75–0.93) | 0.98 (0.971–0.992) |
| PLT | per 10 × 10⁹/L | 0.93 (0.88–0.98) | 0.99 (0.987–0.998) |
| SD | per 10 mm | 1.18 (1.05–1.32) | 1.02 (1.005–1.028) |
| RLT | per 10 mm | 0.69 (0.56–0.85) | 0.96 (0.943–0.984) |
| PVD | per 1 mm | 1.38 (1.16–1.65) | 1.38 (1.157–1.651) |
| Male sex | vs. female | 2.06 (1.12–3.80) | 2.06 (1.118–3.803) |

Estimates are derived from the same model; only the scale of the increment differs. Per-unit odds ratios close to 1.00 for HGB, PLT, SD and RLT reflect the units in which these variables are measured, not a weak association.

**Supplementary Table S14. Intercept recalibration of the HREV-nomogram for settings with a lower expected prevalence of HREV.**

| **Expected local prevalence** | **Original intercept** | **Intercept adjustment** | **Recalibrated intercept** |
| --- | --- | --- | --- |
| 60% | +0.838 | −0.549 | +0.289 |
| 50% | +0.838 | −0.955 | −0.116 |
| 40% | +0.838 | −1.360 | −0.522 |
| 30% | +0.838 | −1.802 | −0.964 |
| 20% | +0.838 | −2.341 | −1.503 |
| 10% | +0.838 | −3.152 | −2.314 |

The six slope coefficients are held fixed and only the intercept is shifted, so that the mean predicted probability matches the expected local prevalence. Adjustments are calculated relative to the training-cohort prevalence of 72.2%; using the full-cohort prevalence of 71.8% changes each adjustment by approximately 0.019 on the logit scale (<0.5% in predicted probability). This table is provided as a starting point for external validation and does not itself constitute validation in a lower-prevalence population.

**Supplementary Table S15. Sensitivity analysis of the outcome definition.**

| **Parameter** | **Primary analysis** | **Sensitivity A: Child-Pugh C-only cases excluded** | **Sensitivity B: Child-Pugh C-only cases counted as non-events** |
| --- | --- | --- | --- |
| Outcome definition | F2/F3, or red signs, or F1 with Child-Pugh C | Medium-to-large varices only | Medium-to-large varices only |
| Analysis set (training / validation) | 403 / 172 | 392 / 162 | 403 / 172 |
| Events (training / validation) | 291 / 122 | 280 / 112 | 280 / 112 |
| AUROC, training | 0.803 | 0.805 | 0.790 |
| AUROC, validation | 0.807 | 0.819 | 0.806 |
| OR male sex | 2.062 | 2.002 | 1.840 |
| OR PVD | 1.382 | 1.401 | 1.372 |
| OR HGB | 0.9818 | 0.9828 | 0.9860 |
| OR PLT | 0.9928 | 0.9929 | 0.9940 |
| OR RLT | 0.9636 | 0.9650 | 0.9697 |
| OR SD | 1.0165 | 1.0174 | 1.0195 |

Because Child-Pugh class contributes to the definition of the outcome, the 21 patients classified as having HREV solely because small varices coexisted with Child-Pugh class C were either excluded (Sensitivity A) or reclassified as non-events (Sensitivity B). Discrimination and all odds ratios were materially unchanged, indicating that the results are not driven by this clause.

**Supplementary Figures**

**Supplementary Figure S1. ROC curves of the HREV-nomogram and the established non-invasive scores in the validation cohort.** The nomogram (AUROC 0.807) is compared with the PC/SD ratio (0.763), FIB-4 (0.737), APRI (0.695) and AAR (0.594). This panel complements Figure 5A of the main text, which shows the training cohort.

**Supplementary Figure S2. ROC curves of individual predictors compared with the full HREV-nomogram in the validation cohort.** Corresponding AUROCs and DeLong P values are given in Supplementary Table S7.

**Supplementary Figure S3. Restricted cubic spline curves relating each continuous predictor to the log odds of HREV.** Three knots were used for each predictor. The likelihood-ratio test for non-linearity was significant only for spleen diameter (P = 0.002); modelling it with a spline did not improve validation discrimination (Supplementary Table S2).

**Supplementary Figure S4. Cross-validation curve and coefficient paths of the LASSO logistic regression.** Left, binomial deviance against log(λ) with λmin and λ1se indicated; right, coefficient paths of the candidate variables. Coefficients are listed in Supplementary Table S4.

**Supplementary Figure S5. ROC curves of the HREV-nomogram by Child-Pugh stratum.** Discrimination was higher in Child-Pugh A patients (AUROC 0.825) than in Child-Pugh B/C patients (0.767);
